# Using Carboxymethyl Cellulose as the Additive With Enzyme-Catalyzed Carboxylated Starch to Prepare the Film With Enhanced Mechanical and Hydrophobic Properties

**DOI:** 10.3389/fbioe.2021.638546

**Published:** 2021-02-02

**Authors:** Can Liu, Shijiao Qin, Jin Xie, Xu Lin, Yunwu Zheng, Jing Yang, Huan Kan, Zhengjun Shi

**Affiliations:** ^1^The Key Laboratory of State Forestry and Grassland Administration on Highly-Efficient Utilization of Forestry Biomass Resources in Southwest China, Southwest Forestry University, Kunming, China; ^2^College of Life Science, Southwest Forestry University, Kunming, China

**Keywords:** carboxymethyl cellulose, amylase, carboxylated starch, composite film, hydrophobicity

## Abstract

Carboxymethyl cellulose, a hydrophobic derivative from cellulose that can be prepared from different biomass, has been widely applied in food, medicine, chemical, and other industries. In this work, carboxymethyl cellulose was used as the additive to improve the hydrophobicity and strength of carboxylated starch film, which is prepared from starch catalyzed by bio-α-amylase. This study investigated the effects of different bio-α-amylase dosages (starch 0.5%, starch 1%) and different activation times (10, 30 min) on starch to prepare the carboxylated starch. The effects of different carboxymethyl cellulose content on the carboxylated starch film were investigated by analysis viscosity, fourier-transform infrared spectroscopy, thermogravimetric analysis, differential scanning calorimetry, x-ray powder diffraction, scanning electron microscope, and contact angle. The results showed that preparing carboxylated starch using activated starch increased the carboxyl content, which could improve the effectiveness of the activated enzyme compared to prolonging the activation time. The carboxyl starch prepared by enzyme catalysis had a lower gelatinization temperature, and enzyme activation destroyed the crystallization area of the starch, thus facilitating the carboxylation reaction. The addition of 15% carboxymethyl cellulose improved the mechanical properties of the prepared film with maximum tensile strength of 44.8 MPa. Carboxymethyl cellulose effectively improved the hydrophobicity of the starch film with the addition amount of 10–30%, while hydrophobic property was stable at 66.8° when the addition amount was exceeded to 35%. In this work, it can be found that carboxymethyl cellulose improve the mechanical and hydrophobic properties of starch film, laying the foundation for the application of carboxylated starch materials.

## Introduction

Cellulose has abundant reserves, low price, environmental protection, and wide application. Carboxymethyl cellulose (CMC), a derivative of cellulose, is a natural anionic water-soluble polysaccharide that is used in food, medicine, chemical, and other industries (Suriyatem et al., 2018; Xie et al., 2018; Biaou et al., 2020). A large number of functional groups, such as carboxyl and hydroxyl, make the technical requirements of the CMC modification process simple, and performance improvement can be achieved with low modification costs. Studies have shown that the addition of CMC can improve the mechanical properties, transparency, and thermal stability of the material (Tasaso, 2015; Huang et al., 2017; Mansur et al., 2017; Kontturi and Spirk, 2019). In the preparation of an electrode film, the addition of CMC can improve the plasticity of the composite film (Ekramulmahmud et al., 2005; Ampaiwong et al., 2019; Nazrin et al., 2020). The above research shows, the CMC has a long carbon chain and polyhydroxyl structure, and is a good scaffold material for hydrophilic material modification. In this paper, CMC is used as the basic material for performance improvement, and the starch film is modified to obtain better material performance.

Starch is the most accessible biomass resource, starch utilization has been a focus of ongoing research. Natural starch has the disadvantages of poor water solubility, low degree of emulsification and gelatinization, and low stability, which limits the application range of natural starch in various industries (Kim et al., 2017; Remya et al., 2018; Escobar-Puentes et al., 2020; Chen et al., 2021). Therefore, modified starches are prepared by introducing new or changing functional groups through physicochemical and biological modifications to equip starch with new characteristics (Sangian et al., 2018; Xiao et al., 2018; Li et al., 2020; Menzel, 2020; Torbica et al., 2020). Because of its strong hydrophilicity and polarity, carboxylated starch has been widely used in drug carriers, adsorbents, flocculants, and other applications (Shahriarpanah et al., 2016; Sarmah and Karak, 2020). Starch is a polycrystalline polymer composed of crystalline, amorphous, and crystalline transition regions (subcrystalline regions) (Sharmin et al., 2020; Wu et al., 2020; Xiao et al., 2020). The modification of starch mostly occurs in the amorphous area within the surface layer of the granules. The unique crystal structure of the crystallization zone makes it difficult for the reagent to penetrate into the starch granules, which limits the further occurrence of chemical reactions, thus resulting in low reaction efficiency and difficulty obtaining products with high substitution degrees (Achremowicz et al., 2000; Shogren, 2000; Cao et al., 2002; Paulik et al., 2019; Shi et al., 2019).

To improve the efficiency of the reaction, starch is often pretreated to destroy its crystalline structure, enhance the degree of reaction, and improve the performance of the modified starch. The current pretreatment methods include physical, chemical, and biological methods (Gong et al., 2017; Zhang et al., 2017; Chang et al., 2019; Holck et al., 2019; Lee et al., 2019). Compared with the other two methods, biological methods are environmentally friendly and mild in response. Based on the characteristics of α-amylase hydrolysis, it was deduced that α-amylase has higher hydrolysis efficiency (Souto et al., 2017; Ozdemir et al., 2018; Wang et al., 2019). Therefore, in the preparation of carboxylated starch, the use of amylase to change the structure of starch granules and reduce the starch crystallization area to improve the reaction efficiency of starch has important theoretical and practical significance. The unique structure of carboxylated starch facilitates water absorption, but the mechanical properties of the material are poor. Therefore, other supportive materials are needed to help improve the performance of carboxylated starch, such as carboxymethyl cellulose.

In order to prepare high value-added carboxylated starch, α-amylase was used to pretreat starch, and then carboxylated starch was prepared. Hope to destroy the crystalline structure of starch to a greater extent. Films were prepared using carboxylated starch, Carboxymethyl cellulose was used to improve the hydrophobicity and mechanical properties of the carboxylated starch films. The carboxyl content, Fourier-transform infrared spectroscopy (FTIR), thermogravimetric analysis (TGA), differential scanning calorimetry (DSC), x-ray powder diffraction (XRD), scanning electron microscope, contact angle, mechanical properties, and other parameters were used to characterize the prepared carboxylated starch and film. For the first time, the effect of enzyme pretreatment on the performance and application of carboxylated starch was studied. At the same time, the effect of CMC on starch film was discussed to establish a foundation for the industrial application of carboxylated starch film.

## Materials and Methods

### Experimental Materials

Starch-Unmodified was purchased from Sigma-Aldrich, USA. Sodium hypochlorite, active chlorine≥7.5%, AR grade, purchased from China Shanghai Titan Technology Co., Ltd. Potassium iodide test paper was purchased from China Shanghai Sanaisi Reagent Co., Ltd, and coppe sulfate pentahydrate was purchased from China Shanghai Titan Technology Co., Ltd., AR grade. Sodium hydroxide was purchased from China Shanghai Titan Technology Co., Ltd., AR grade. Hydrochloric acid sodium hydroxide was purchased from China Shanghai Titan Technology Co., Ltd., AR grade, purity 36~38%. Carboxymethyl cellulose hydroxide was purchased from China Shanghai Titan Technology Co., Ltd., M.W.90000(DS = 0.7), AR grade. Sodium sulfite was purchased from Shanghai Titan Technology Co., Ltd., China, AR grade, SO_2_ ≥ 98.0%. According to GB/ T601-2002 chemical reagent solution standard, 0.4% copper sulfate solution, 4% sodium hydroxide solution, 3% hydrochloric acid solution, 0.1 mol/L hydrochloric acid solution, 0.085 mol/L sodium hydroxide solution and 10% sodium sulfite were prepared.

### Preparation Process

#### Amylase Pretreatment

According to the data and preliminary test results, the optimized process was: Toke 60 g of native starch in a round bottom flask and added 90 g of water to made 4 groups of 40% starch milk, put the flask in a water bath and heated to 55°C and adjusted pH = 6. This study investigated the effects of different bio-α-amylase dosages and different activation times on starch to prepare the carboxylated starch. After the enzymolysis reaction was completed, used sodium hydroxide to adjusted the pH=9 to kill the enzyme. After the sample is dried and crushed, waiting to be used in the next step. The samples 1: enzyme content 0 g, activation 0 min; sample 2: enzyme content 0.3 g, activation 10 min; sample 3: enzyme content 0.6 g, activation 10 min; sample 4: enzyme content 0.3 g, activation 30 min; sample 5: enzyme content 0.6 g, activation 30 min.

#### Preparation Process of Carboxylated Starch

The starch milk pretreated by the first step enzyme was added into a round-bottom flask with agitator and temperature control device, and the pH was adjusted to 9 by 3% sodium hydroxide and 3% hydrogen chloride. Increased the temperature to 45°C, added 15 g NaClO (15% of dry starch), kept the constant temperature for reaction, after reaction for a period of time, determined the pH value in the reactor, and neutralized with 3% HCl, the reaction time was 6 h. After the reaction, potassium iodide test paper was used to determine whether the reaction of the modifier NaClO was complete. Added an appropriate amount of 10% sodium sulfite solution to stopped the reaction, toke out the reaction flask, washed the product with distilled water for three times, then put it in the oven for 4 h at 45°C, and then grinded the carboxylated starch.

#### Preparation of Starch Film

Weighed 4 g carboxylated starch, preparation of 4% starch aqueous solution. and weighed 0 g, 0.2 g, 0.4 g, 0.6 g, 0.8 g, 1 g, 1.2 g, 1.4 g, 1.4 g, 1.4, g and 1.6 g carboxymethyl cellulose in conical flask (the addition amount of carboxymethyl cellulose was 0, 5, 10, 15, 20, 25, 30, 35, 40% of carboxylated starch), and dissolved with water. The solution was mixed in a conical flask and placed on a magnetic agitator. The rotating speed was adjusted to make the rotor rotate at a constant speed. After dissolution, vacuum degassing, and defogging were carried out. After completion, the solution was poured into the glass culture dish, drying in 45°C drying oven for 6 h to peel off the film.

### Characterization and Analysis

Starch samples were prepared into 6% modified starch milk, heated in a water bath at 95°C for 15 min, and gelatinization temperature was measured by NDJ-5S rotary viscosimeter of Shanghai Pingxuan Scientific Instrument Co., LTD; FTIR used magnair 560E.S.P infrared spectrometer from Nicolet Company of The United States. The potassium bromide pressure plate/ATR method was adopted to scan the range of 4,000 to 400 cm^−1^, and the number of scans was 64; DSC used DSC204 differential scanning calorimeter manufactured by NETZSCH, Germany. The sample was 3.5, and 5 mg of water was added with a micro sampler. The program was set at 5°C/min, 200°C was the termination temperature, and argon gas was blown and protected at 30 mL /min; TG-DTG was used by TGA209 F3, NETZSCH, Germany. The samples were dried in a drying oven at 50°C for 24 h, weighed 3~5 mg, and the heating rate was 10°C/min. Argon was used as protective gas, and thermogravimetric analysis was performed on the samples at a gas flow rate of 30 ml/min; XRD test adopted Japanese Neo-D/MAX220, the optical tube was made by Philips and the target material was Cu. The sample was 120 mesh powder, dried at 100°C for 8 h. The test conditions were as follows: voltage 40 kV, current 30 mA, starting angle of 10°, termination angle of 60°, step width of 0.02°, curve fitting peak parting method was adopted for calculation (Chen et al., 2020); SEM was determined by Quanta200 SCANNING electron microscope from Dutch FEI Company. The observed environment was vacuum, the surface of the sample was treated with gold spray; The contact angle tester of Kruss DSA produced by Kruss DSA of Germany was used to test the surface wettability of the film sample; the carboxyl content was measured by the following method: 5 g samples were placed in a 150 mL beater, 25 mL 0.1 mol/L HCl solution was added, the samples were stirred for 30 min, filtered (filtered) with glass sand core funnel, washed with non-amino distilled water (distilled water cooled after boiling), and tested for chloride ion free with silver nitrate solution. The carboxylated starch after ash removal was transferred to a 600 mL beaker, 300 mL distilled water was added, heated and boiled (5~7 min), and NaOH solution was used for calibration.

## Results and Discussion

### Enzyme's Effection on Carboxylated Starch

**Figure 1:**
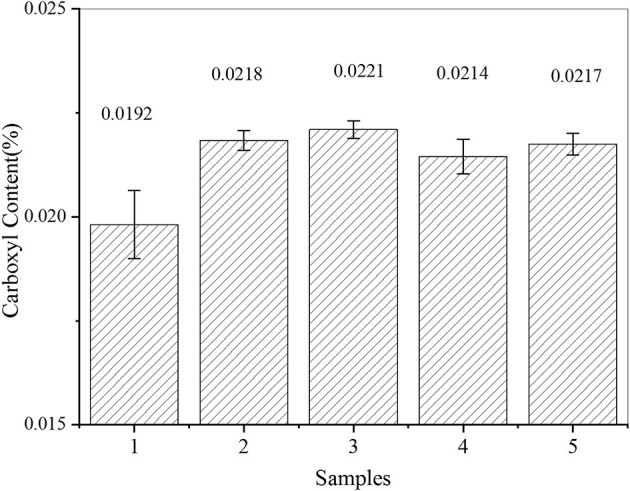
Trend chart of the starch carboxyl content.

In this part of the experiment, five levels of experiments were designed using five samples. The carboxyl content was determined for each of the five samples. According to the analysis shown in Figure 1, the carboxyl group content of carboxylated starches prepared by the enzyme activation treatment was higher than that of native starch. The enzyme treatment effectively improved the carboxylation activity. On the basis of the same enzyme activation time, the amylase dosage increased the carboxyl content of modified starch. The rule was that with a greater the amount of enzyme, the carboxyl content of modified starch would be higher. Alpha-amylase is an endonuclease, but it is difficult to break the crystalline shell of starch granules. The activation may have been caused by the entry of amylase into the interior through the pores of starch granules and its reaction with the semi-crystalline soft shell, which increased the carboxyl content. Due to the limited pore quantity, the increased amount of enzyme only increased the carboxyl content for a limited time, it is unclear in what way the increase was limited (Ma et al., 2006; Salcedo-Mendoza et al., 2018; Navia-Coarite et al., 2019; Zeng et al., 2019). Using the same enzyme dosage, prolonging the activation time of amylase reduced the starch carboxyl content, but the degree of change was not significant.

**Figure 2:**
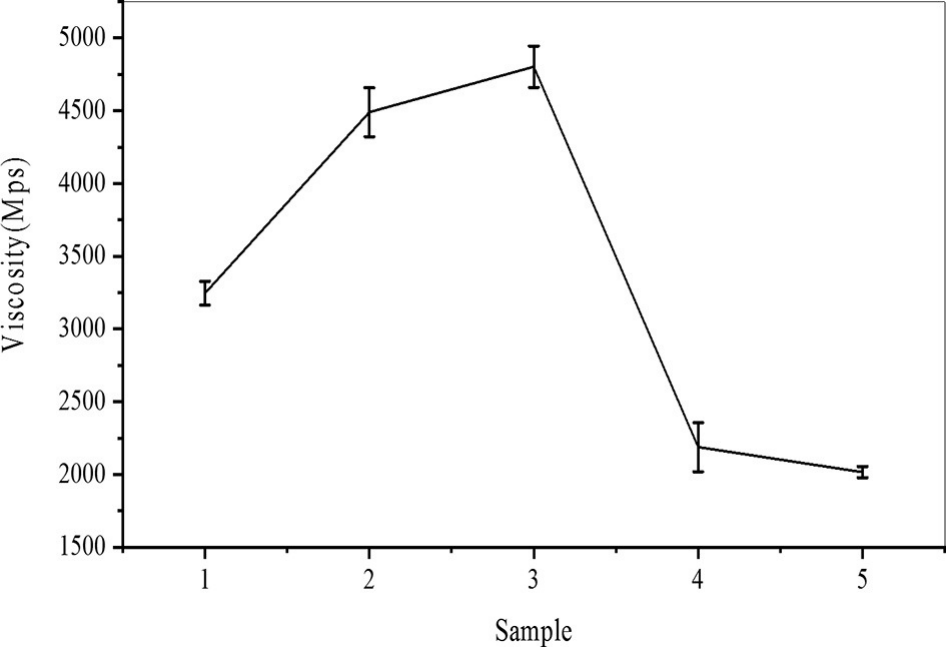
Viscosity trend chart of carboxylated starch.

Viscosity is an index of the molecular weight of carboxylated starch. The decrease in starch viscosity was caused by a decrease in the degree of polymerization and molecular weight of starch macromolecules due to the carboxylation agent, which led to a decrease om gelatinization viscosity. It can be seen from Figure 2 that the viscosity of the sample after 30 min of enzyme activation was lower than that of the other three samples, and sample five had the lowest viscosity. It can be seen that extending the enzyme activation time can effectively reduce the molecular weight of starch. At the same activation time, the amount of enzyme had little effect on the viscosity of starch, which may be due to the limited number of pores on the surface of starch granules. Therefore, once the maximum threshold is exceeded, the amount of enzyme added has little effect on the viscosity of the modified starch. Hence, the amount of enzyme has little effect on the molecular weight. Compared with the addition of the enzyme, the viscosity of modified starch was effectively reduced by prolonging the activation time.

**Figure 3:**
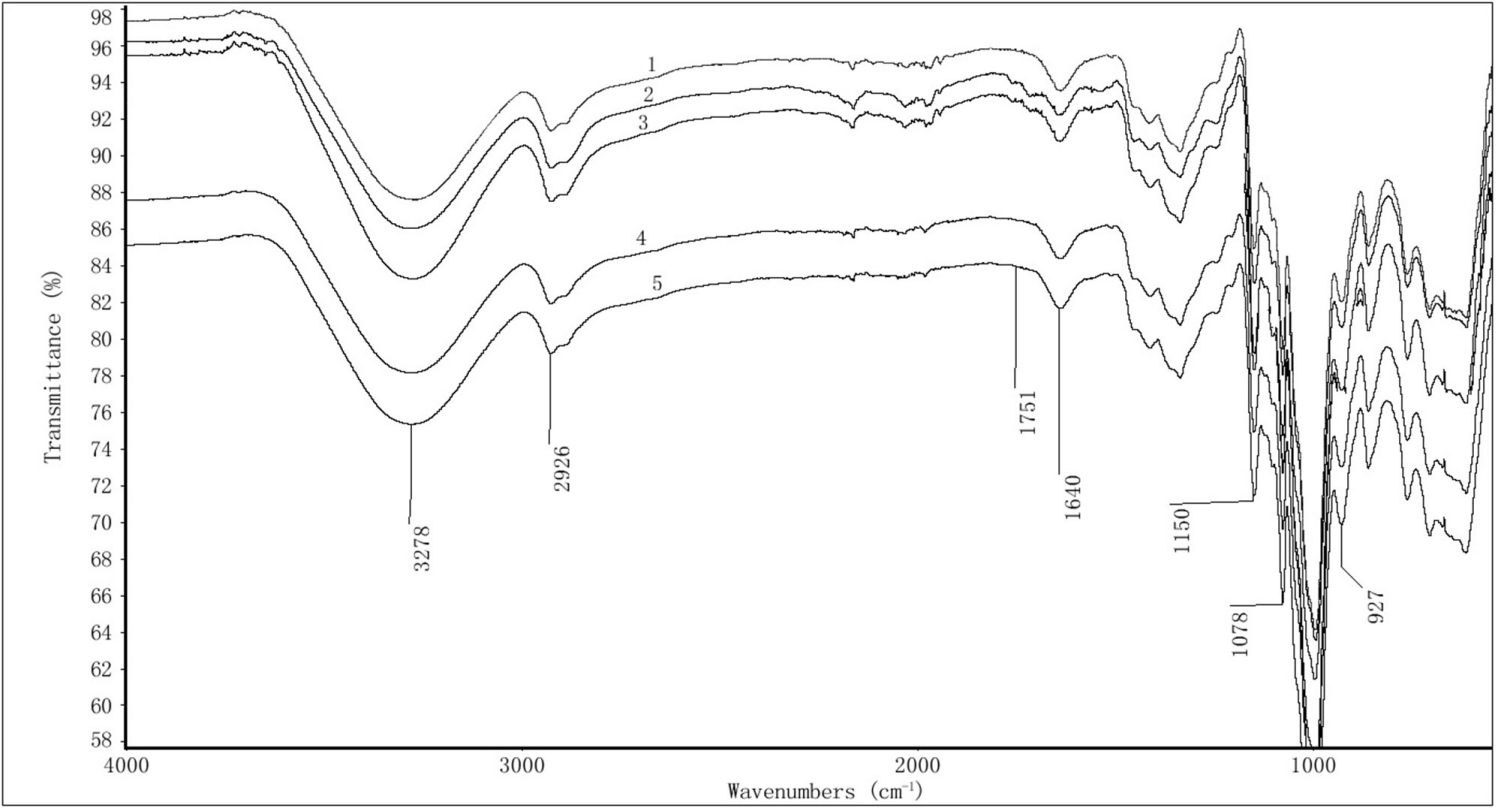
FTIR diagram of carboxylated starch.

As shown in Figure 3, starch is composed of D-anhydroglucose units as basic units, and the most important characteristic groups are the hydroxyl groups on C_2_, C_3_, and C_6_. Among them, C_2_ and C_3_ were secondary alcoholic hydroxyl groups, and C_6_ connected the primary alcoholic hydroxyl groups with a D-pyran ring structure. The positions of the infrared absorption peaks and structural assignments of these characteristic groups in the infrared spectrogram were as follows: The hydrogen-bonded O-H stretching vibration occurred near 3,278 cm^−1^; the O-H asymmetric stretching vibration occurred near 2,928 cm^−1^; the H-O-H bending vibration that occurred near 1,640 cm^−1^ was caused by water, and many scholars mistakenly believe via a starch analysis that it was the absorption peak of C=O; the C-O-C asymmetric stretching vibration peak occurred near 1,150 cm^−1^; the C-O stretching vibration of the D-glucopyranose band connected to the hydroxyl group occurred near 1,078 cm^−1^; and the glycosidic bond vibration occurred at 927 cm^−1^ as. Carboxylated starch not only contains the absorption peak of the above characteristic groups, but also a C=O absorption peak near 1,751 cm^−1^. Therefore, the occurrence or non-occurrence of a peak near 1,751 cm^−1^ determines whether the carboxylated starch contains carboxyl and carbonyl groups. It can be seen from Figure 3 that the C=O absorption peak at 1,751 cm^−1^ can only be clearly observed in samples 2 and 3. However, this peak cannot be observed in the other levels. It can be seen that the degree of carboxylation of starch after enzyme activation was higher, and the FTIR data is consistent with the carboxyl content detection data.

**Figure 4:**
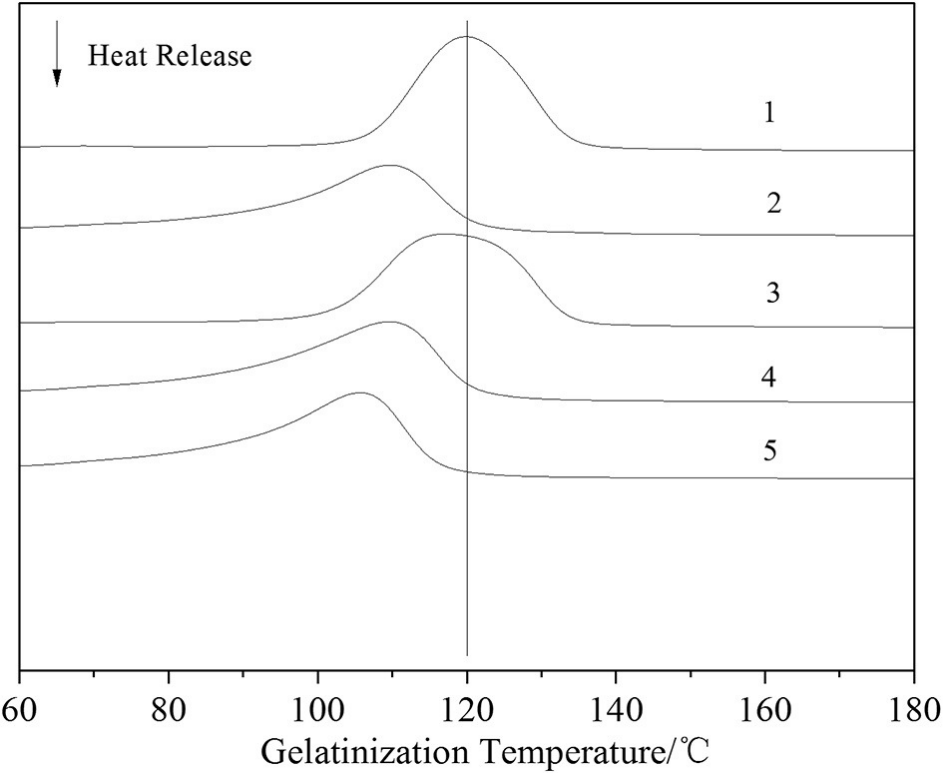
DSC analysis of carboxylated starch.

Five starch samples were analyzed using DSC, the starch gelatinization start temperature (To/°C), starch gelatinization peak temperature (Tp/°C), gelatinization end temperature (Tc/°C), and heat absorbed by gelatinization (ΔH/J·g^−1^). It can be seen from Figure 4 and Table 1 that the gelatinization temperature of carboxylated starch was lower than that of native starch. The analysis showed that the activation of the enzyme opened and expanded the pores leading to the inside of the starch granule. Thus, the crystallization area was destroyed during carboxylation preparation, which made it easier for water to enter the inside of the modified starch granule and, in turn, lowered the gelatinization temperature of the carboxylated starch compared to that of native starch. At the same time, the gelatinization enthalpy of the carboxylated sample decreased compared with that of the original starch. It is also proved that enzyme-activated starch was more easily destroyed by carboxylation.

**Table 1:** DSC analysis of carboxylated starch.

**Sample**	***T*p/°C**	***T*o/°C**	***T*c/°C**	**ΔH/J·g^**−1**^**
1	119.9	107.4	133.6	1368
2	109.7	94.2	120.7	973.8
3	117.2	103	133.4	1409
4	109.6	90.21	120.7	1252
5	105.8	90.4	116	1184

**Figure 5:**
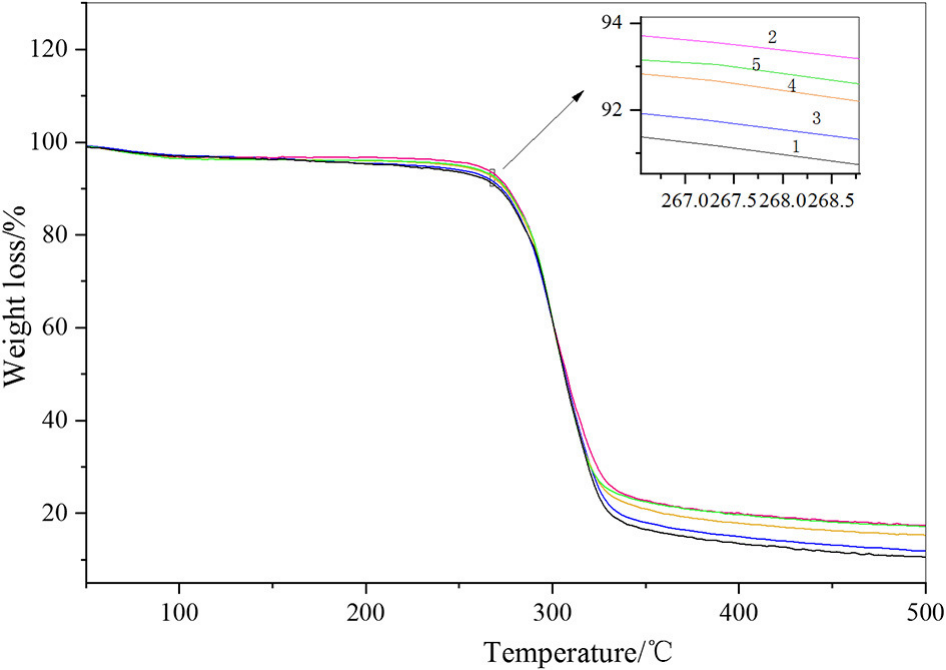
TGA analysis of carboxylated starch.

It can be seen from Figure 5 and Table 2 that the TGA curves of the four carboxylated starch samples were essentially the same as those of the original starch. It can be clearly seen that the TGA curve of starch is divided into two stages of weight loss. The first weight loss stage occurred at ~100°C, and the second weight loss stage occurred at ~300°C. The first weight loss stage occurred due to the evaporation of water, and water in starch consists of two parts. The first part contains free water molecules, and the second part was the water that bound to the starch hydroxyl groups. The first part of water was easily lost, whereas the second part was difficult to lose. The free water in the starch sample started to volatilize at ~60°C and ended at ~150°C. The second part contained less water, and the temperature when the loss occurred was generally above 200°C. Due to the low water content of the hydroxyl group, no obvious weight loss peak could be observed in the TGA analysis. It can be clearly seen that the weight loss rate of the original starch was higher than that of the other carboxylated starch samples. The bound water in carboxylated starch was destroyed during the oxidation reaction process, which resulted in the bound water of carboxylated starch being less than that of starch, which is one reason why the weight loss rate of carboxylated starch was less than that of starch. The thermal degradation temperature of the carboxylated starch sample increased slightly, and the unstable structure of the starch reduced in size during the oxidation reaction. The carboxylated starch sample was soluble in water during the washing process, and while the remaining structure was more compact and stable, the thermal stability performance was enhanced (Pei et al., 1985; Jha, 2020).

**Table 2:** TGA analysis value of carboxylated starch.

**Sample**	**Starting temperature** **(**°**C)**	**End temperature** **(**°**C)**	**Weight loss rate** **(%)**
1	280.9	326.1	75.35
2	281.5	320.6	73.96
3	279.2	324.1	74.79
4	286.4	321.2	73.91
5	281.6	321.6	73.34

According to Table 3, the X-ray diffraction patterns of the five samples were essentially the same, with single peaks occurring near 15(2), 19(2), and 23(2), and a crystalline double peak occurring near 17(2) (Supplementary Figure 1). The carboxylated starch prepared by enzymatic activation has a similar pattern to that of natural starch. According to Table 3, the crystallinity of the four carboxylated starch samples was lower than that of the original starch, which indicates that enzyme activation promoted the carboxylation reaction of starch, destroyed the starch crystallization area, and reduced the crystallinity of starch. At the same activation time, the greater the amount of enzyme that was used, the more seriously the crystal zone was destroyed and, thus, the lower was the crystallinity. The relative activation time and enzyme dosage had a significant influence on the crystallinity of starch.

**Table 3:** XRD analysis of carboxylated starch.

**Sample**	***I*(cps)**	***Ia*(cps)**	***I*c(cps)**	***X*c(%)**
1	51816.46	36856.46	14960.00	28.87
2	50683.36	37034.87	13648.50	26.93
3	49685.30	36698.13	12987.17	26.14
4	48677.63	35252.40	13425.24	27.58
5	49987.40	36944.26	13043.14	26.09

### Preparation of Composite Films

**Figure 6:**
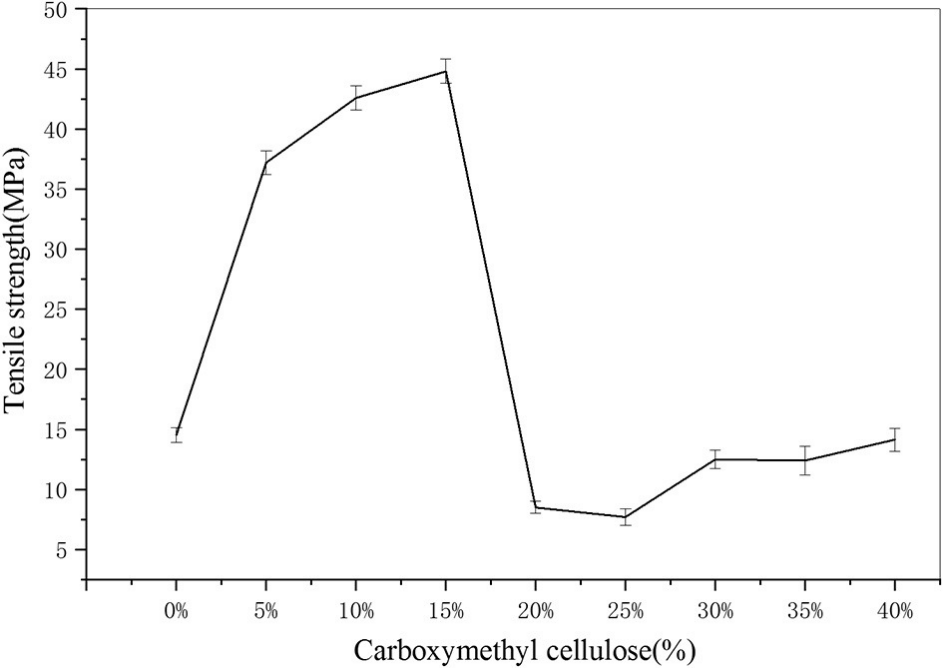
Tensile strength of the film.

As can be seen from Figure 6, with the increase of carboxymethyl cellulose, the tensile strength of the film first increased, reaching its maximum tensile strength at 44.8 MPa when the cellulose content was 15%, and then decreased with further increases of cellulose, the mechanical properties of the films declined significantly.

**Figure 7:**
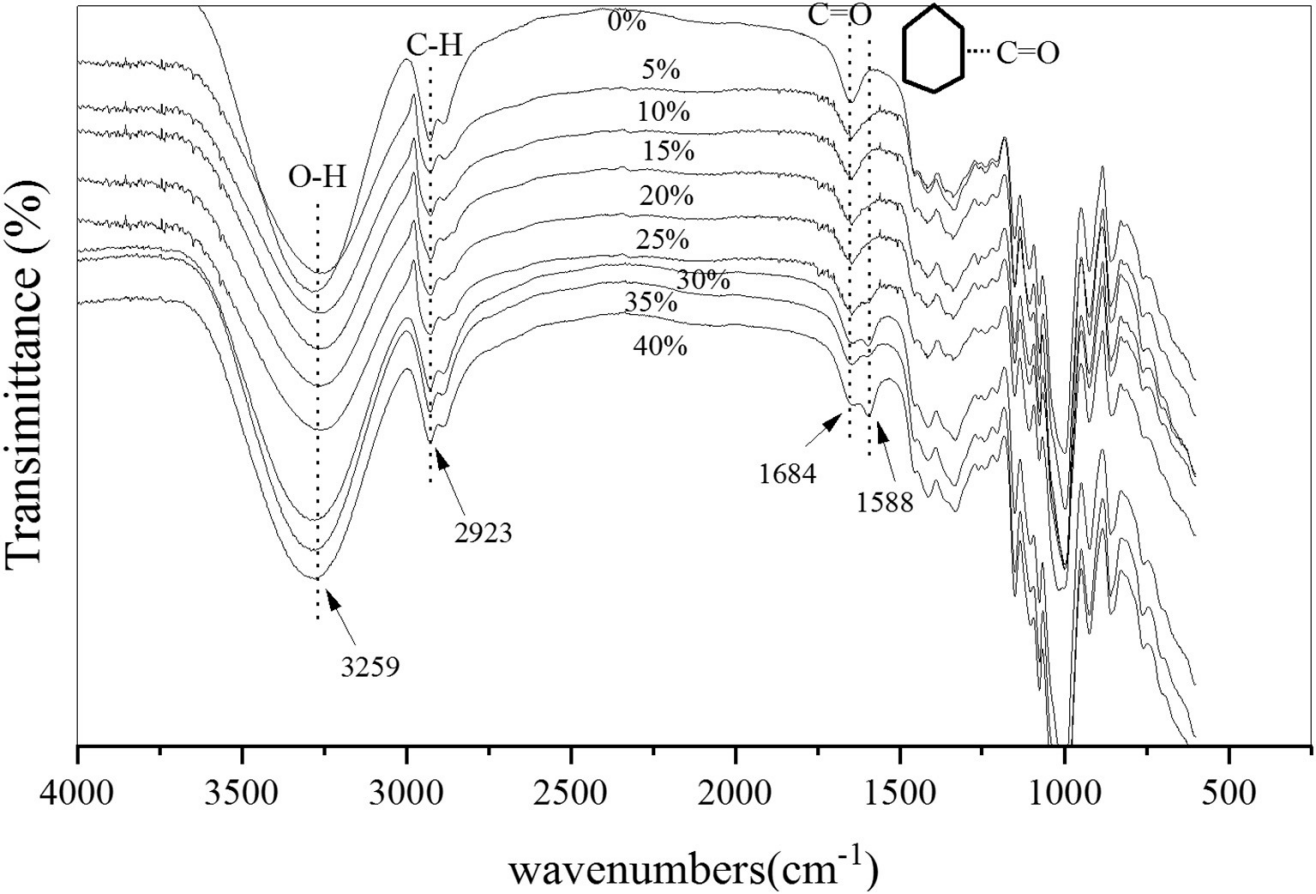
Infrared spectroscopic analysis of the film.

It can be seen from Figure 7 that the FTIR data showed more starch characteristics. The positions of the infrared absorption peaks and the structural assignments of these characteristic groups were, respectively, in the infrared spectrogram: The stretching vibration of hydrogen-bonded (O-H) occurred at ~3,259 cm^−1^; the asymmetric stretching vibration of C-H occurred at ~2,923 cm^−1^, and, the absorption peak of C=O occurred at ~1,684 cm^−1^. The asymmetric stretching vibration peak of C-O-C occurred at ~1,148 cm^−1^; the C-O stretching vibration of the D-glucopyranose band connected to the hydroxyl group occurred at ~1,075 cm^−1^; and the glycosidic bond vibration occurred at ~918 cm^−1^. With the increase of cellulose <30%, the characteristic peaks at ~3,259 cm^−1^, ~2,923 cm^−1^, and ~1,075 cm^−1^ slightly deviated but did not change significantly. When the amount of cellulose was >30%, a new peak appeared at ~1,588 cm^−1^, and the peak increased with the increased amount of cellulose, which through analysis was determined to be the vibration of the aromatic C=O skeleton. With the increase of the amount of cellulose addition, the carboxyl content of cellulose increased along with its reaction probability with the hydroxyl group in starch. Some starch and cellulose groups produced an esterification reaction and formed an aliphatic aromatic ring. Thus, the aromatic C=O skeleton vibration appeared in the FTIR diagram (Da Silva Filipini et al., 2020; Li and Wei, 2020; Zhou et al., 2020).

**Figure 8:**
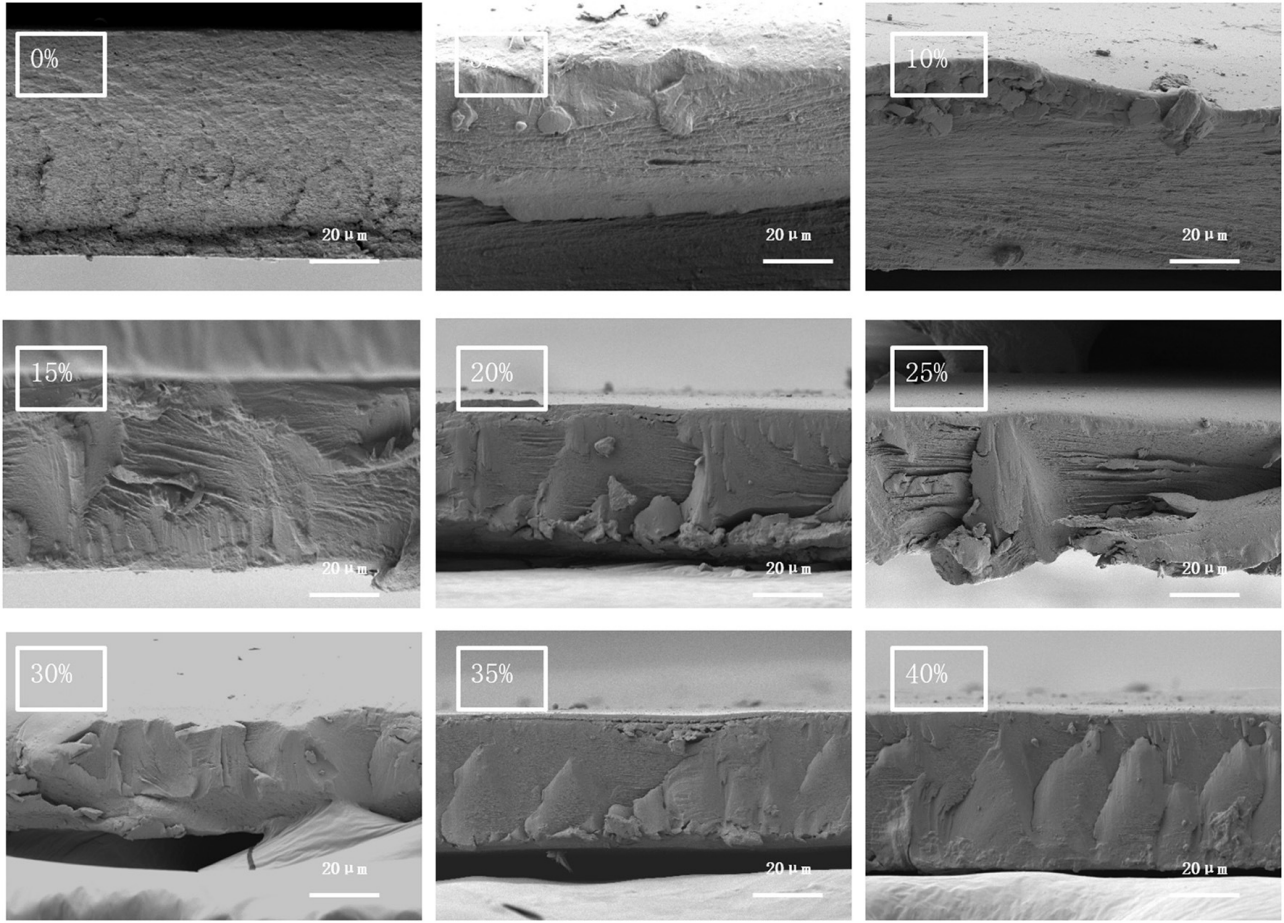
Electron micrograph of the film section.

It can be seen from Figure 8, through the cross-sectional electron micrograph, it could be seen that the cross-section of the film was not smooth, and the graininess was particularly strong when the cellulose addition was zero. With the increase of the cellulose addition, the cross-sectional particle morphology of the film decreased and gradually showed fiber morphology. A blocky smooth section appeared when the addition amount was >20%, and a blocky break appeared in the film section when the addition amount was >30%. The analysis showed that as the addition amount increased, the fiber agglomerated and gradually separated from the starch. As carboxymethyl cellulose tends to be non-polar, phase separation occurred when the two materials were not closely bound, and the two materials were disconnected at the junction where they were not closely connected. Combined with the mechanical property analysis of the film, it was determined that when the additive amount exceeded 15%, the mechanical property of the film began to decline.

**Figure 9:**
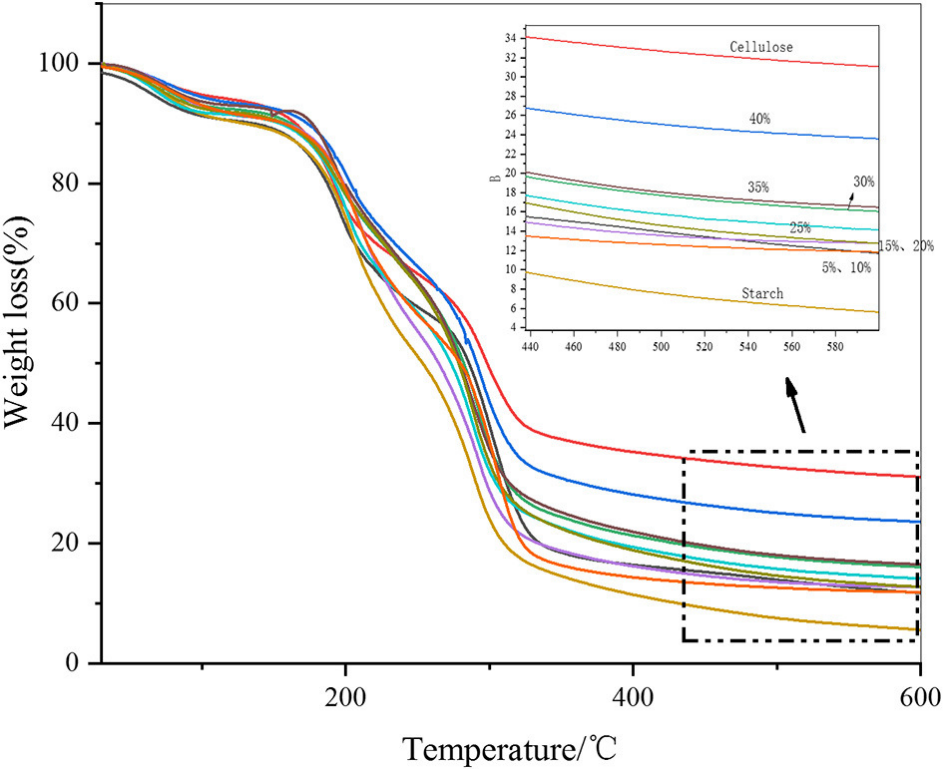
Thermogravimetric analysis of the films.

As shown in Figure 9, when the fiber additive amount was zero, the weight loss rate of the film was highest, and the residual carbon rate was the lowest at 5.6%. The highest carbon yield was 23.6% when 40% cellulose was added. With the increased cellulose content, the overall carbon yield of the film increased. The thermal properties of the two films were similar when the additive amount was 5 and 10%. When the additive amount was 15 and 20%, the properties of the two materials were similar. In general, the addition of cellulose helped to improve the thermal stability of the film materials.

**Figure 10:**
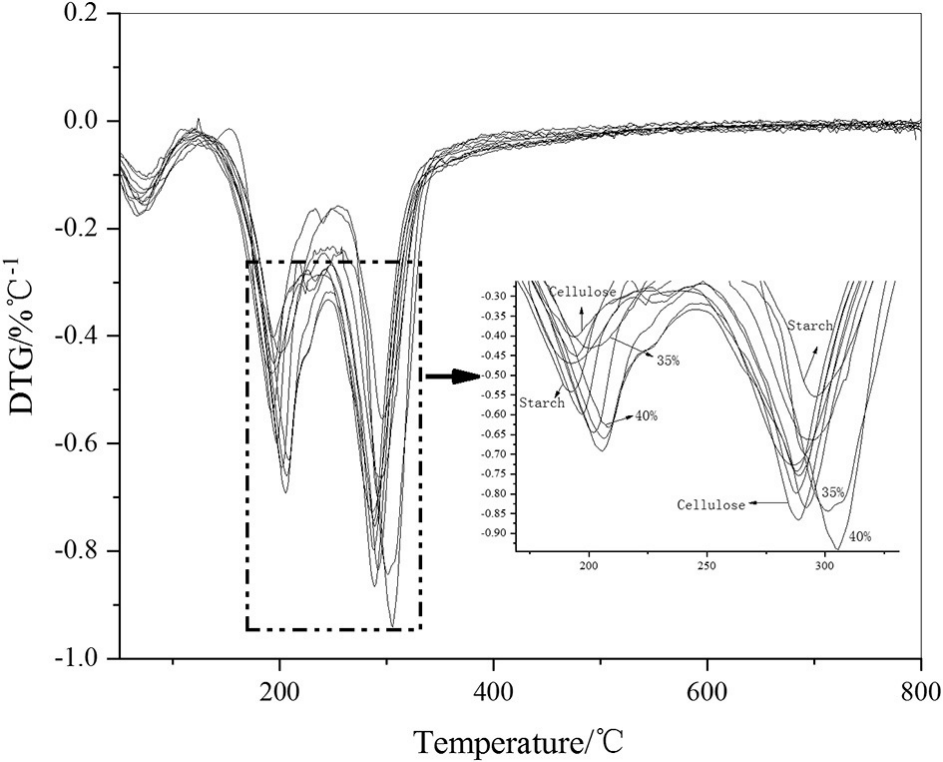
DTG diagram of the film.

A derivative thermogravimetry (DTG) graph was developed to differentiate the TG data of the thin film materials. In Figure 10, two thermal decomposition peaks can be clearly seen. The first decomposition peak occurred at ~200°C, which was the thermal decomposition peak of starch. The second peak occurred at ~300°C, which was the thermal decomposition peak of cellulose. Because there is a large amount of high crystallinity amylose in starch, some of it was decomposed at 300°C. Similarly, the structure of non-oriented cellulose in cellulose was similar to that of starch, which decomposes at ~200°C. However, from a general point of view, with the addition of cellulose, the 200°C decomposition peak of the film material was delayed, which indicated that the starch structure reacted with cellulose and the thermal stability was enhanced. Similarly, at ~300°C, the thermal stability of cellulose was enhanced due to the highly crystallized starch, and the pyrolysis peak was also delayed. The thermal stability was highest when the additive amount reached >35%, which exceeded the structural performance of the raw material itself. Combined with infrared analysis, it can be seen that due to the increased amount of cellulose, cellulose and starch formed an aromatic ring. Infrared data showed that an aromatic C=O skeleton vibration appeared at ~1,588 cm^−1^. The thermal performance of the aromatic structure increased with respect to the temperature of the aliphatic structure.

**Figure 11:**
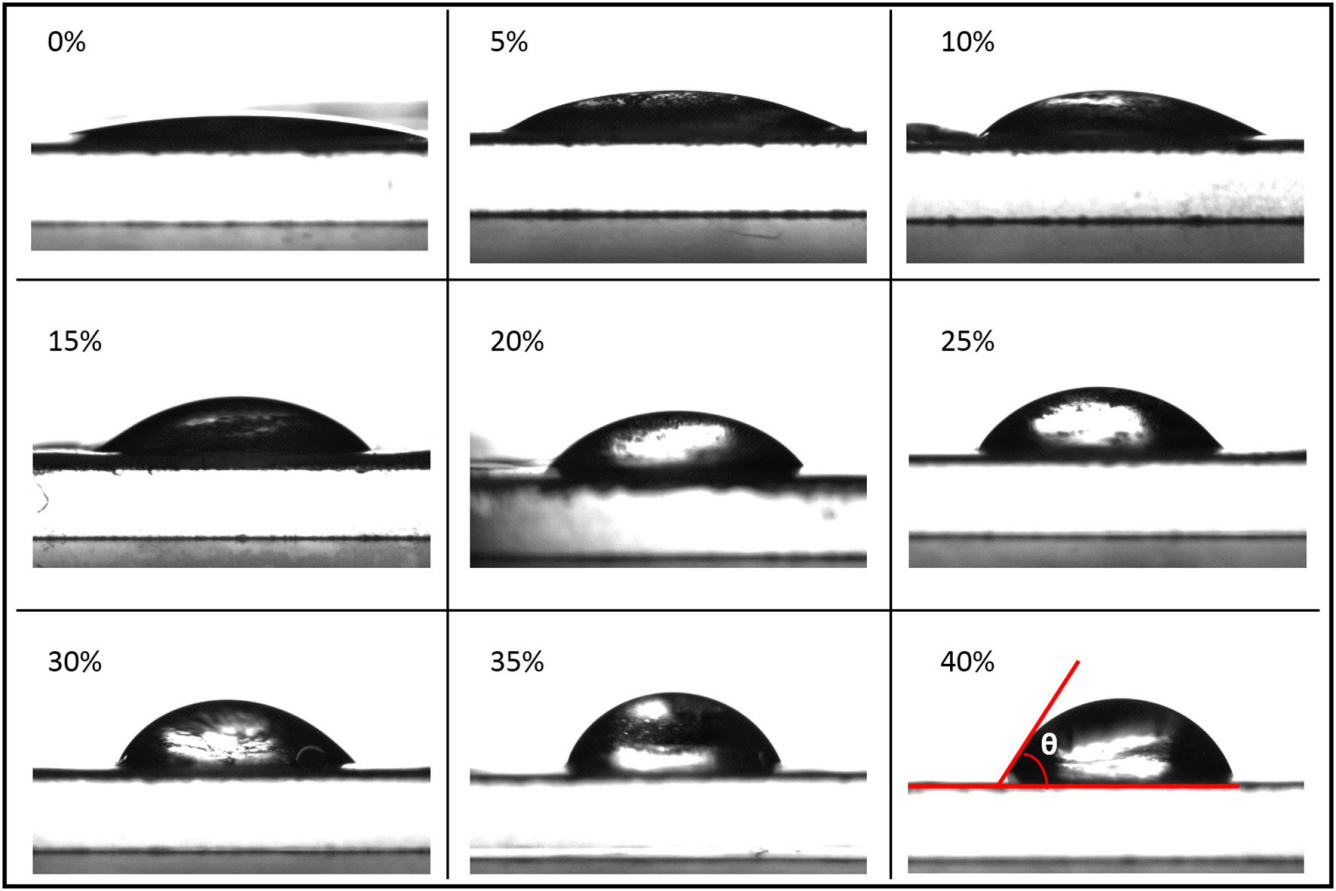
Contact angle diagram of the film.

**Figure 12:**
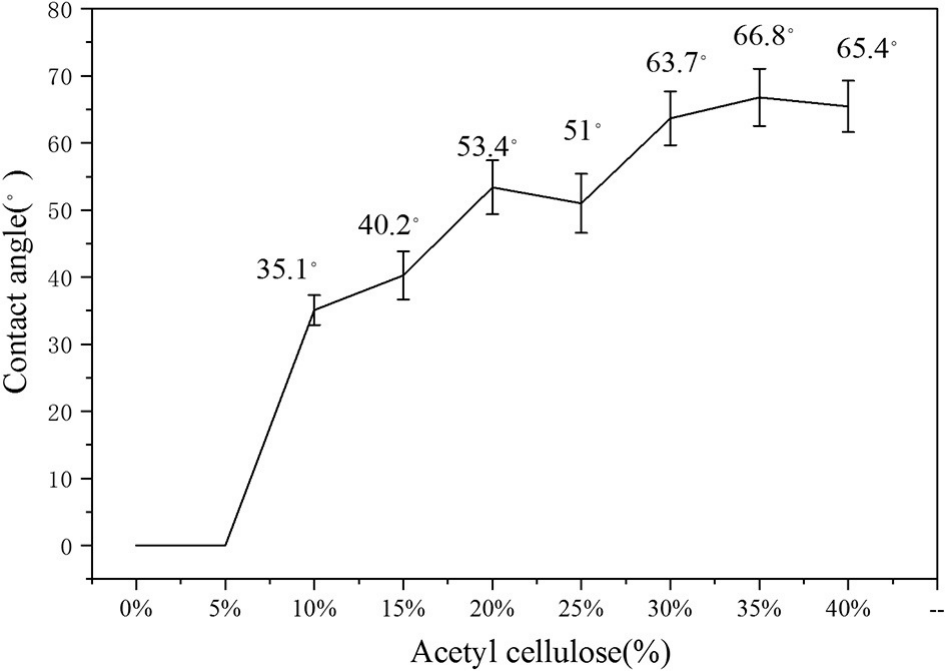
Trend diagram of the film contact angle.

It can be seen from Figures 11, 12 that the film contact angle increased with the increase of cellulose addition. However, the film contact angles of 0 and 5% could not be measured. The analysis showed that starch, which is a water-absorbing material, and the carboxylated modified starch film had better water absorption, which caused water to be absorbed by the film in a short time, and the contact angle could not be measured. The film contact angle was improved when the additive amount was >10%. It can be clearly seen from the data, that 10, 20, and 30% were the three turning points when the contact angle improved. When the added carboxymethyl cellulose was >10%, the starch film was significantly improved. The analysis showed that because the hydroxyl groups on the starch structure were oxidized to the carboxyl groups, the water absorption of the starch film greatly improved. A small amount (<5%) of the addition did not increase the distribution of cellulose on the surface, and it had a minor effect on the contact angle of the film. The addition of >10% significantly improving the contact angle. The increase in the contact angle was not significant when more than 30% cellulose was added. The analysis showed that the cellulose material was rich in hydroxyl groups and easily absorbed water. When >30% cellulose was added, the characteristics of the cellulose material limited further improvement to the hydrophobicity. The hydrophobic effect of the film was best when 35% cellulose was added, and the contact angle was 66.8%.

## Conclusion

In conclusion, carboxymethyl cellulose was used as the additive to improve the hydrophobicity and strength of carboxylated starch film, which is prepared from starch catalyzed by bio-α-amylase. Activated enzyme was more effective at increasing the carboxyl content than was prolonging the activation time of the enzyme. Enzyme activation destroyed the crystalline structure of starch and effectively promoted the carboxylation reaction. Carboxymethyl cellulose effectively improved the hydrophobicity of the starch film with the addition of >10% carboxymethyl cellulose. The contact angle was 66.8° when the additive amount was 35%. The addition of carboxymethyl cellulose improved the mechanical properties of the membrane material, and the maximum membrane performance was 44.8 MPa when 15% was added. The addition of carboxymethyl cellulose can form an aliphatic aromatic structure partly through an esterification reaction, which can improve the thermal stability of thin film materials. The modified carboxylated starch film can be used at higher temperature and humidity, expanding the application range of the film.

## Data Availability Statement

The original contributions presented in the study are included in the article/Supplementary Material, further inquiries can be directed to the corresponding author/s.

## Author Contributions

CL, HK, and ZS conceived the project and contributed to the concept of the manuscript. SQ, JX, and XL synthesized and characterized the carboxylated starch, and performed experimental works. JY and YZ synthesized and characterized the composites. All authors contributed to the article and approved the submitted version.

## Conflict of Interest

The authors declare that the research was conducted in the absence of any commercial or financial relationships that could be construed as a potential conflict of interest.
