# Supplementary material for: Using Carboxymethyl Cellulose as the Additive With Enzyme-Catalyzed Carboxylated Starch to Prepare the Film With Enhanced Mechanical and Hydrophobic Properties
Source: Front Bioeng Biotechnol. 2021 Feb 2;9:638546. doi: 10.3389/fbioe.2021.638546 (PMC7884610; doi:10.3389/fbioe.2021.638546)
Supplement: Supplementary file 1 [file Image_1.pdf]

Supplementary Information

Using carboxymethyl cellulose as the additive with enzyme-catalyzed carboxylated starch to prepare the film with enhanced mechanical and hydrophobic properties

Can Liu[a], Shijiao Qin[b], Jin Xie[a], Xu Lin[a], Yunwu Zheng[a], Jing Yang, Huan Kan [b]* and Zhengjun Shi[a]*

a The Key Laboratory of State Forestry and Grassland Administration on Highly-Efficient Utilization of Forestry Biomass Resources in Southwest China，Southwest Forestry University, Kunming 650224, PR China

b College of Life Science, Southwest Forestry University, Kunming, 650224, PR China

Supporting Figures

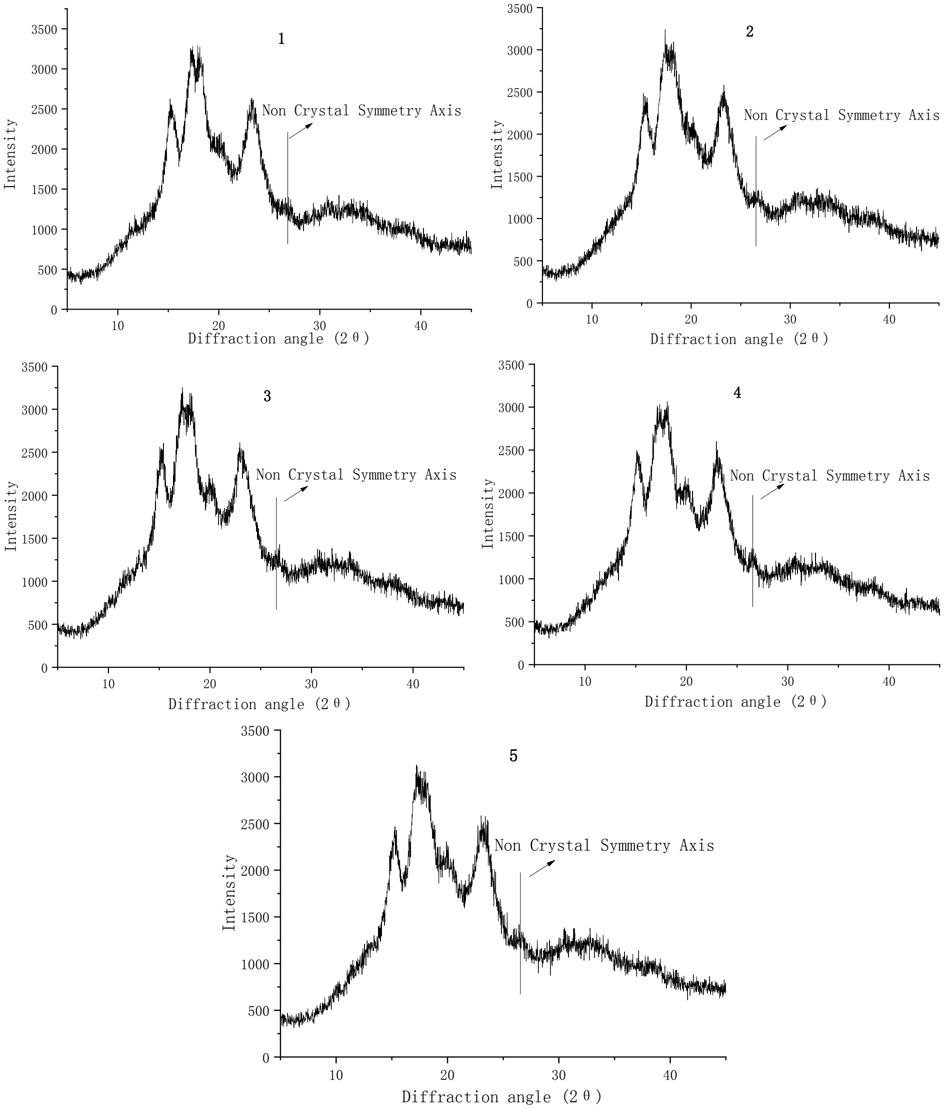

Figure S1 X-ray diffraction pattern of starch
